# Body Mass Index and Sedentary Behaviour Affect Hamstring Extensibility in Primary Education Students

**DOI:** 10.3390/sports13040109

**Published:** 2025-04-07

**Authors:** Guillermo De Castro-Maqueda, Miguel Ángel Rosety-Rodríguez, Jorge R. Fernández-Santos

**Affiliations:** Department of Physical Education, School of Education Science, University of Cádiz, 11519 Puerto Real, Spain; miguelangel.rosety@uca.es (M.Á.R.-R.); jorgedelrosario.fernandez@uca.es (J.R.F.-S.)

**Keywords:** flexibility, physical activity, body mass index, sit and reach test, deep trunk flexion test

## Abstract

Enhancing and maintaining physical condition is an essential element of physical education for primary school children. In this respect, flexibility is of crucial importance in physical performance and coordination. One of the areas addressed in physical education is that of increasing hamstring flexibility, which is diminished by physical inactivity and inversely associated with the body mass index. The objective of this study is to explore the interplay between hamstring extensibility, physical inactivity and overweight in primary school students. Accordingly, a cross-sectional descriptive study was carried out of 265 students, applying sit and reach (SR) and deep trunk flexion (DTF) tests and analysing responses made to the IPAQ questionnaire. Among the results found for the study sample, the average flexor capacity recorded was −0.26 ± 6.33 among the boys and 5.52 ± 6.46 among the girls. The highest values in the girls were achieved at 9–10 years of age (6.69 ± 8.19) and in the boys at 6–7 years of age (2.72 ± 5.99). The lowest values for the girls (3.30 ± 5.19) were recorded in those aged 8–9 years and for the boys (3.13 ± 5.60) at the same age. These study results suggest there is a relationship between a sedentary lifestyle, a high BMI and sub-optimum flexibility. The children identified as physically active obtained higher average results in both the SR and the DTF tests.

## 1. Introduction

Physical fitness is an essential component of overall health and its development during childhood represents a critical period for establishing motor competencies and health-related habits that can persist into adulthood [1]. Flexibility is a key component of physical fitness as it allows for an adequate range of joint motion. Additionally, chronic static stretching exercises have the potential to enhance muscle strength and power by addressing muscle–tendon restrictions [2,3]. Specifically, hamstring muscle extensibility is crucial for maintaining proper posture, performing fundamental movements and preventing musculoskeletal discomfort [4,5]. Despite its importance, flexibility has traditionally received less attention than other components of physical fitness, such as cardiovascular endurance or muscular strength. In school settings, physical education programs often prioritise other fitness dimensions, potentially overlooking the long-term benefits of improving flexibility [6,7].

In recent decades, the growing prevalence of physical inactivity and sedentary behaviour among children has raised concern due to the associated negative implications for health and physical development [8]. Factors such as increased screen time, reduced outdoor play and urbanised lifestyles have collectively contributed to a marked decline in children’s mobility and flexibility [9]. Concurrently, childhood overweight and obesity have reached alarming global proportions, with considerable increases reported in many regions with high levels of social and economic development [10]. Physical inactivity and excess body weight are closely interrelated and their combined impact on muscle extensibility is becoming increasingly evident. Excess adiposity may alter joint biomechanics and increase mechanical load, thereby limiting children’s ability to achieve and maintain adequate hamstring flexibility [11,12].

The evidence suggests that low hamstring flexibility is associated with reduced participation in physical activities both within and outside the school environment [9,13]. Reduced time in structured physical education, along with a rise in sedentary pastimes, such as playing video games or watching television, have been linked to declines in flexibility among school-aged children [13]. Additionally, overweight and obesity may further compromise muscle and tendon properties, potentially reducing tissues’ adaptability to stretching [12]. The cyclical nature of these factors—limited flexibility, physical inactivity and overweight—highlights the need for comprehensive strategies that integrate movement-based activities, stretching routines and healthy weight management within educational settings.

Regular engagement in physical activity that includes stretching exercises can significantly improve hamstring extensibility and other key musculoskeletal traits [14]. Without appropriate interventions, insufficient flexibility may exacerbate musculoskeletal problems, back pain and movement limitations that can persist into adolescence and adulthood [1]. Flexibility is less indicative of health than other fitness components. Moreover, lifestyle factors, such as motivation and self-perception of ability, play a role in whether children choose to engage in activities that could enhance flexibility, especially among those with excess weight who may experience discomfort or fear of negative judgment [11].

The originality of this study lies in its comprehensive analysis of the triadic relationship between flexibility, inactivity and overweight within the school environment. By identifying key factors that influence flexibility in children, this research seeks to provide evidence-based recommendations for the development of structured school programs that integrate flexibility training alongside other fitness components.

The purpose of this study is to explore the interplay between hamstring extensibility, physical inactivity and overweight in primary school students, considering its implications for educational and public health stakeholders. Specifically, we hypothesise that children with lower levels of physical activity and a higher body mass index (BMI) will exhibit reduced hamstring flexibility, reinforcing the need for targeted interventions.

## 2. Material and Method

### 2.1. Participants

The initial sample population for this cross-sectional study consisted of 273 Caucasian primary school children aged 6–12 years (137 boys and 136 girls) recruited in a random order. However, eight failed to meet the inclusion/exclusion criteria and were excluded from the analysis, leaving a final study population of 265 children. The exclusion criteria were the presence of acute lumbar pain (3 children), musculoskeletal injury in the leg (3 children), or a previously diagnosed structural spinal injury (2 children). The participants were instructed not to perform any physical exercise in the 24 h prior to the measurement session in order to avoid possible distortions.

The sample was divided into the following sub-samples by grade (school year): first grade (n = 43), second grade (n = 43), third grade (n = 43), fourth grade (n = 47), fifth grade (n = 44) and sixth grade (n = 45).

All participants and their parents or guardians were informed about the protocol of the study and the experimental risks and benefits of participation. The children gave their assent and their parents/guardians gave signed consent for their children to take part in this research. The children were also informed of their right to refuse participation in the study at any time. None of the participants or their parents/guardians reported any discomfort during the performance of this research. The study was conducted in accordance with the principles of the Declaration of Helsinki (Fortaleza, Brazil, October 2013), with the International Council on Harmonisation Guidelines for Good Clinical Practice and the Spanish legal framework for clinical research on humans (Royal Decree 561/1993 on clinical trials). Moreover, the study was approved in 2023 by the University of Cadiz Ethics Committee for Research Involving Human Subjects at the request of the principal investigator, who took all the measurements. The researcher’s qualifications included a Ph.D. in Sports Medicine and a bachelor’s degree in Physical Education, demonstrating expertise in both the medical and pedagogical aspects of sports sciences.

### 2.2. Measures

The measurements were taken during two consecutive weeks in April 2023. Each participant had only one day of testing, which was performed during a timetabled physical education class and under the same conditions in every case (see procedures).

*Body mass index* (BMI). Height and body mass were measured with the participants barefoot, wearing shorts and a t-shirt. Height was measured to the nearest 0.1 cm using a stadio meter (Holtain, Pembrokshire Crymmych, UK), and body mass was assessed to the nearest 0.1 kg using a Seca scale (SECA GmbH& Co. KG, Hamburg, Germany). All instruments were calibrated to ensure acceptable accuracy. BMI was calculated as body mass/height squared (kg/m^2^) and categorised in line with the BMI international cut-off values as underweight (UW), normal weight (NW), overweight (OW), or obese (OB) [15,16].

*Hamstring flexibility*. This component was evaluated using the sit and reach test (SRT) following the protocol described by Ayala, Sainz de Baranda, De Ste Croix and Santoja [17]. It has high validity and reliability [18,19] and is among the most commonly used linear methods [4,20]. In the SR test, initially described by Wells and Dillon [21], the participants were instructed to sit on the floor, legs together and extended and feet flexed at 90° against a measurement box marked with a scaled ruler (PO Box 1500, Fabrication Enterprises Inc., White Plains, NY, USA). The participants were evaluated wearing sportswear (shirt and shorts) and no shoes. Then, with the palms of both hands downwards and the fingers outstretched, they were told to advance as far as possible, sliding their hands along the ruler, and to hold this position for at least two seconds. The SR test score (in cm) was recorded as the final position of the fingertips on or towards the ruler. Higher scores indicated greater flexibility. The test was performed twice, and the best score was retained [21,22,23,24,25,26].

In addition, the deep trunk flexion (DTF) test was performed, following the protocol described by Zurita et al. [26] and as used previously [26,27,28] to evaluate flexor capacity, determined by the changes in spinal posture during deep anterior flexion of the trunk. For the DTF test, the participants adopted a standing position, barefoot and legs apart, on a wooden platform (0.76 by 0.88 m), with the heels of both feet parallel to a line indicating the value 0 on the scale (marked in cm). They were then told to flex their knees and extend their hands between their legs, reaching as far back as possible. This extension was recorded on the ruler.

*Physical activity:* The level of physical activity was determined using the International Physical Activity Questionnaire (IPAQ, 2011) [29], which, among other items, asks respondents about the number of episodes and duration (minimum 10 min) of physical activity in the last seven days. According to the information provided, each participant was classified as physically active or inactive.

### 2.3. Procedure

Before performing the aforementioned tests, body composition was measured. The participants then performed a standard warm-up consisting of 5–10 min of aerobic running, followed by two sets of standardised static stretching exercises, each lasting 30 s [23,24,25].

Bouncing was not allowed, and the participants were told to perform the stretch slowly and calmly. The best result obtained from each test was used for statistical analysis. The tests were conducted in a covered sports hall, always at the same time of day and under the same environmental conditions, at a room temperature of 22–24 °C.

### 2.4. Sample Size Estimation

Sample size calculations were performed using G*Power software version 3.1v (University of Düsseldorf, Düsseldorf, Germany). According to the computation of the required sample size for the t-test for means differences between two independent samples, a sample of 138 participants is needed to obtain a significant difference between genders (required input parameters: effect size = 0.5, level of significance = 0.05, power = 0.80 and allocation ratio = 1). Regarding the between-subjects ANOVA with interaction terms, a sample of 247 participants is needed to obtain a significance result (required input parameters: effect size = 0.5, level of significance = 0.05, power = 0.80 and number of groups = 12 (6 grades × 2 genders)).

### 2.5. Data Analysis

Means and standard deviations were calculated for the quantitative data and counts and percentages for the categorical data. Gender differences were assessed using an independent samples *t*-test for the quantitative data and the chi-square test for the categorical data. A between-subjects ANOVA model was fitted with grade and gender as factors, and physically active and *BMI* were included as covariates. Additionally, a post hoc analysis was conducted to test all the pairwise comparisons between genders across grades. The Bonferroni correction was applied to adjust for the increased risk of Type I error associated with multiple pairwise comparisons. Standardised residuals of the fitted models were checked for normality using the Shapiro–Wilk test of normality. A significance level of α = 0.05 was set for hypothesis testing, and a partial eta-squared was used to estimate the effect size in the ANOVA model. All analyses were conducted using the R programming language for statistical computing (version 4.2.2).

## 3. Results

### 3.1. Participants’ Characteristics

The study subjects were 129 male and 136 female primary school children aged 6–12 years. Table 1 summarises their characteristics for the group as a whole and classified by age and by grade (school year). The mean (±SD) BMI values recorded were 18.5 ± 3.4 for the whole group, 18.6 ± 3.5 for the girls and 18.4 ± 3.3 for the boys.

### 3.2. Hamstring Flexibility

Overall, the girls performed better than the boys in both flexibility tests (*p* < 0.001) despite the boys’ greater level of physical activity (*p* < 0.05). In the DTF test, the girls scored 24.2 ± 4.8, and the boys scored 24.2 ± 4.8. In the SR test, the girls scored 5.5 ± 6.5, and the boys scored −0.5 ± 6.6 (see Table 1).

### 3.3. Physical Activity

According to the between-subjects ANOVA, the variables physically active, grade and gender are significantly associated with the outcomes of both tests (Table 2). However, BMI was only a significant predictor in the DTF test (*p* < 0.001).

**Figure 1:**
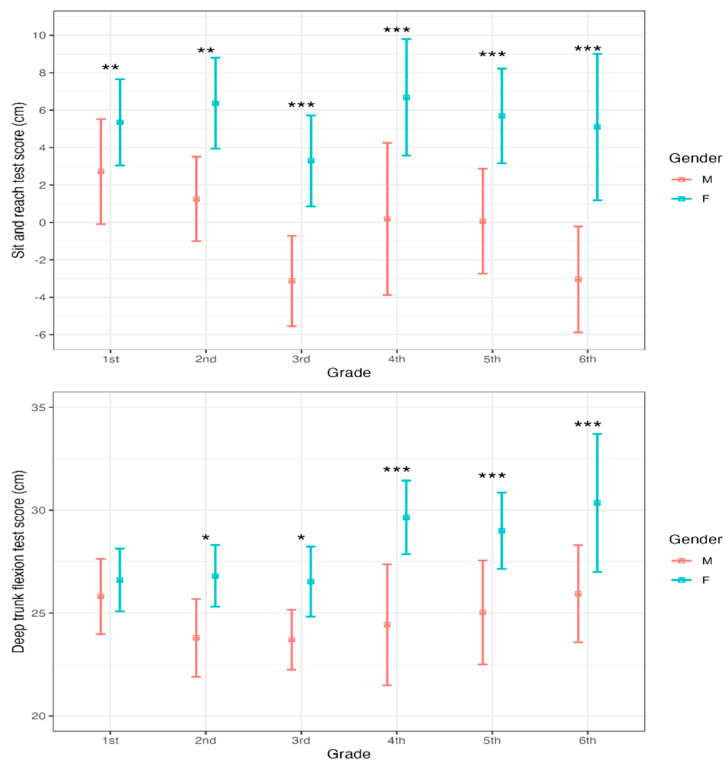
Gender differences in the DTF and SR tests by school grades. * *p* < 0.05; ** *p* < 0.01; *** *p* < 0.001.

Post hoc pairwise comparisons show that significant differences were observed in both tests across all grades (school years) for the SR and DTF tests, except in first grade for the DTF test (Figure 1). 

## 4. Discussion

This study examined the relationship between hamstring flexibility, body mass index (BMI) and physical inactivity in primary school children. The results indicate that girls demonstrated significantly greater flexibility than boys, as measured by sit and reach (SR) and deep trunk flexion (DTF) tests. This difference could be attributed to gender-related factors, reflecting possible biological, hormonal and sociocultural differences in the development of this physical quality [1,14]. Additionally, children classified as physically active exhibited better flexibility outcomes compared to their inactive counterparts, highlighting the positive influence of movement-based activities on musculoskeletal function. Furthermore, a higher BMI was associated with lower flexibility scores, suggesting that excess body weight may contribute to reduced muscle extensibility and joint mobility [24].

The multifaceted relationship between hamstring extensibility, physical inactivity and overweight in children underscores the complexity of addressing health and fitness in early life. Beyond its association with posture and musculoskeletal health, hamstring flexibility is also linked to broader aspects of physical literacy, motor skill development and overall functional movement quality [30]. Limited flexibility can hinder children’s ability to perform fundamental movement skills, which are critical for engaging in various forms of exercise and sports participation [9]. As children grow and mature, differences in growth velocity, biological maturation and body composition can influence joint range of motion and tendon stiffness, further affecting flexibility outcomes [15,31].

This disparity, which has been documented in various contexts, underscores the need for tailored educational interventions aimed at improving muscle extensibility according to sex, school grade and fitness level [5,32].

The interplay between overweight and reduced flexibility is not merely a biomechanical issue. Excess adiposity often alters gait patterns and provokes postural adaptations, raising energy expenditure in basic movements and potentially discouraging regular physical activity due to discomfort or perceived difficulty [11,12]. Such challenges can contribute to a downward spiral of decreasing activity, lower fitness levels and an increased risk of future health complications. Interventions that foster physical activity and address weight management can help break this cycle. For instance, school-based physical activity programs, which often include aerobic exercises, skill development and structured stretching, have demonstrated their effectiveness in improving various fitness parameters, including flexibility [23,24]. These approaches should form part of comprehensive frameworks that consider growth, maturation and readiness for training, ensuring that activities are age-appropriate and safe.

Moreover, interventions focusing on fundamental movement skills, such as balance, coordination and body awareness, may indirectly improve flexibility by increasing overall muscular engagement and joint mobility. Research has shown that children who develop robust motor skills at a young age are more likely to remain physically active, achieve a healthy body weight and maintain positive perceptions of exercise throughout adolescence [1,9]. Dietary education and family engagement also play important roles in preventing overweight and obesity, facilitating better weight status and indirectly promoting conditions conducive to improved muscle extensibility [27].

Longitudinal data also suggest that these outcomes are influenced by population-level trends. For example, secular declines in physical fitness components have been documented in school children, reflecting broader environmental and lifestyle changes [33]. These trends reinforce the necessity of sustained, evidence-based policies and interventions at multiple levels—individual, family, school and community—to enhance hamstring flexibility, reduce sedentary behaviour and address childhood overweight. Comprehensive strategies integrating physical education curriculum reforms, extracurricular sports, active transport to school and community-based recreation programs can foster a supportive environment for maintaining and improving children’s physical flexibility [24,26].

The sit and reach test, initially described by Wells and Dillon [21], has become a reference method for assessing hamstring and lumbar flexibility due to its simplicity, accessibility, reliability and validity [17,18,19,20]. Ayala et al. [17], among others, have consolidated its utility across different populations. Other researchers have emphasised its importance in school settings, highlighting its ability to detect functional deficits and correlate flexibility with factors such as physical activity, overweight and motor performance [18,19,20,23,34]. Incorporating the SR test into physical education programs enables practitioners not only to establish flexibility profiles but also to evaluate the impact of pedagogical strategies aimed at improving joint mobility and preventing muscle imbalances.

In practice, the value of simple, valid measures like the SR test is that they enable regular, scalable monitoring of flexibility status [18,19,20]. Combined with other field tests and anthropometric assessments, they provide educators and health professionals with a holistic understanding of a child’s musculoskeletal health. This information can guide tailored interventions that not only improve flexibility but also enhance overall physical fitness, motor competence and psycho-social well-being, ultimately laying the foundation for healthier, more active lifestyles lasting into adolescence and beyond [18,34,35].

Complementing the SR test, the deep trunk flexion (DTF) test, used in several previous studies [24,25,26], offers an integrated perspective on spinal mobility. The DTF test examines changes in spinal curvature during flexion, providing additional information on trunk functionality. Together, these tests provide a more comprehensive evaluation of flexibility, identifying restrictions associated both with the hamstring muscles and spinal mobility—critical aspects in preventing lower back pain, maintaining proper posture and ensuring the efficient execution of daily movements [26,27].

A substantial body of literature consistently indicates that lack of physical activity, sedentary behaviour, increased screen time and overweight all have a negative impact on children’s flexibility [5,10,11,12]. Numerous studies have demonstrated that prolonged inactivity reduces the range of joint motion and muscle extensibility, impairing physical performance and predisposing children to muscle injuries at early ages [24,33,36]. Excess weight, in turn, imposes greater mechanical loads on the lower limbs, altering the biomechanical properties of tissues and limiting their ability to elongate. This situation is further compounded when children with reduced flexibility perceive increased discomfort during physical activity, thus reinforcing a vicious cycle of sedentariness and overweight [1,5,8].

Regular exercise, especially if it involves activities that incorporate stretching, is associated with substantial improvements in hamstring extensibility, trunk mobility and a reduced risk of injury [24,25,26,27,28]. Structured school-based interventions, community programs and physical education curricula that include regular stretching sessions can enhance neuromuscular function, body composition and exercise motivation [5,9,32]. Recent research has demonstrated that the implementation of multidisciplinary programs—encompassing nutritional education, playful activities and systematic stretching—can optimise flexibility and overall fitness in school children. Developing more extensive motor competencies not only improves flexibility but also enhances confidence, academic performance and the propensity to adopt active lifestyles during adolescence and adulthood [37,38,39].

Addressing overweight through strategies to prevent childhood obesity—focusing on reducing sedentary behaviour and on promoting healthy eating habits and regular physical activity—has positive repercussions on flexibility and other markers of physical fitness [11,12,16,40]. The literature shows that early interventions in school and family environments can prevent the emergence of deficient movement patterns, correct postural alterations and foster adherence to sports practice [38,40]. These actions are particularly relevant because flexibility is an auxiliary factor in preventing back pain, improving posture and optimising performance in multiple sports disciplines [4,14,24,25].

The systematic inclusion of tests such as the SR and the DTF in schools, in addition to serving as diagnostic tools, enables long-term monitoring of the effects of pedagogical and health interventions. In response to the multifactorial complexity surrounding the development of flexibility, integrating these tests with other indicators (body composition, level of physical activity, exercise motivation and nutritional status) provides a holistic view of children’s health and fitness [1,5,10,11,32].

Promoting hamstring extensibility in children is best understood as part of a broader, integrated effort to combat inactivity and overweight. By implementing multifaceted, school-based and community-driven initiatives that encourage regular physical activity, develop fundamental motor skills and support healthy body composition, we can improve children’s physical flexibility and overall health outcomes. Such approaches would empower them to engage more confidently in physical activities, break cycles of inactivity and lay the groundwork for a healthier future.

In short, implementing well-structured school programs that address physical fitness holistically, including targeted flexibility exercises, is crucial to success in this regard. Such interventions can yield multifaceted benefits, including improving overall functional capacity, enhancing self-esteem and fostering positive attitudes towards lifelong physical activity [5,13]. Ultimately, the goal of this investigation is to guide the development of evidence-based school interventions in support of flexibility and encourage healthy, active lifestyles that are essential for children’s overall well-being [41].

This research, supported by an extensive body of literature, confirms the negative impact of physical inactivity and overweight on hamstring and lumbar flexibility in primary school children. Conversely, it shows that sustained and multidisciplinary interventions, curricular adjustments and the promotion of adapted physical activity routines can reverse these trends, improving flexibility and contributing to children’s overall health. In view of these findings, we believe it imperative to consolidate educational and health policies that integrate flexibility, weight management and regular exercise, thereby ensuring optimal physical development and enhanced quality of life for future generations.

## 5. Potential Interventions to Improve Flexibility

Given the observed relationship between flexibility, physical activity and BMI, targeted interventions could be implemented to enhance children’s hamstring extensibility and overall mobility. School-based physical education programs should incorporate structured stretching routines, dynamic flexibility exercises and movement-based activities to improve muscle elasticity and prevent musculoskeletal discomfort. For instance, integrating yoga, Pilates and proprioceptive neuromuscular facilitation (PNF) stretching techniques into school curricula has been shown to enhance flexibility and motor control in children.

In addition, promoting active recess periods and extracurricular sports participation can provide children with additional opportunities to engage in movement-based activities that contribute to increased flexibility. Encouraging parents to facilitate outdoor play and limit sedentary screen time could also support flexibility development outside the school environment. Moreover, targeted interventions for overweight children, such as combined physical activity and nutritional education programs, could help mitigate the negative impact of excess body weight on flexibility by improving overall fitness levels and promoting healthier lifestyle habits.

## 6. Limitations and Future Research Directions

Despite its contributions, this study has several limitations that should be acknowledged. First, the cross-sectional design precludes the ability to establish causal relationships between physical inactivity, BMI and flexibility outcomes. Longitudinal studies are needed to assess how these variables interact over time and determine whether specific interventions yield sustained improvements in flexibility.

Second, while the SR and DTF tests are widely used measures of flexibility, they primarily assess static flexibility and may not fully capture dynamic movement patterns relevant to daily activities and sports performance. Future research could incorporate additional functional assessments to provide a more comprehensive evaluation of muscle extensibility.

Furthermore, while the IPAQ questionnaire provided valuable insights into children’s physical activity levels, self-reported data may be subject to recall bias and social desirability effects. Future studies could integrate objective measures such as accelerometery to enhance the accuracy of physical activity assessments.

Lastly, future investigations should explore the potential psychosocial factors influencing children’s participation in flexibility-enhancing activities, such as motivation, self-efficacy and perceived enjoyment. Understanding these elements could help refine intervention strategies and promote long-term adherence to movement-based practices.

In conclusion, this study highlights the importance of physical activity and weight management in maintaining optimal hamstring flexibility in primary school children. Implementing targeted interventions that integrate flexibility exercises within school and community settings could yield significant benefits for children’s musculoskeletal health and overall physical well-being.

## 7. Conclusions

This study demonstrates that flexibility is inversely related to BMI and positively associated with physical activity in primary school children. Among the study population, girls consistently showed better flexibility than boys. Promoting healthy weight management and regular physical activity is essential to optimise flexibility and overall fitness in children. These findings underscore the need for targeted interventions in school-based physical education programs to combat the adverse effects of obesity and inactivity on flexibility.

Furthermore, inactivity is related to the increase in overweight in this age group as a consequence of poorer hamstring and lower back flexibility.

## Figures and Tables

**Table 1:** Characteristics of the sample.

	All (n = 265)	Girls (n = 136)	Boys (n = 129)
Height (cm)	137.3 ± 11.2	137.1 ± 11.0	137.5 ± 11.9
Weight (kg)	35.7 ± 11.4	35.6 ± 11.1	35.6 ± 11.3
BMI (kg/m^2^)	18.5 ± 3.4	18.6 ± 3.5	18.4 ± 3.3
DTF (cm)	26.6 ± 5.1	28.2 ± 4.8	24.8 ± 4.8 ***
SR (cm)	2.6 ± 7.2	5.5 ± 6.5	−0.5 ± 6.6 ***
Physically active (n [%])			
Yes	136 [51%]	60 [44%]	76 [59%] *
No	129 [49%]	76 [66%]	53 [41%]

BMI, body mass index; DTF, deep trunk flexion test; SR, sit and reach test. * *p* < 0.05. *** *p* < 0.001.

**Table 2:** Multivariate ANOVA results.

	DTF		
Variables	F	*P*	$\eta_{p}^{2}$ (90% CI)
BMI	37.641	**<0.001**	0.130 (0.072–0.196)
Physically active	24.864	**<0.001**	0.090 (0.041–0.150)
Grade	6.269	**<0.001**	0.111 (0.045–0.162)
Gender	53.875	**<0.001**	0.177 (0.111–0.247)
Grade × Gender	1.320	0.256	0.026 (0.000–0.048)
	SR		
Variables	F	*P*	$\eta_{p}^{2}\;$ (90% CI)
BMI	2.593	0.109	0.010 (0.000–0.041)
Physically active	154.316	**<0.001**	0.381 (0.306–0.449)
Grade	2.553	**<0.05**	0.048 (0.003–0.082)
Gender	121.795	**<0.001**	0.327 (0.252–0.397)
Grade × Gender	1.069	0.378	0.021 (0.000–0.040)

DTF, deep trunk flexion test; SR, sit and reach test.

## Data Availability

The data that support the findings of this study are available from the corresponding author, G.D.C.-M. upon reasonable request. The data are not publicly available due to privacy and ethical restrictions.
